# Top Mysteries of the Mind: Insights From the Default Space Model of Consciousness

**DOI:** 10.3389/fnhum.2018.00162

**Published:** 2018-04-24

**Authors:** Ravinder Jerath, Connor Beveridge

**Affiliations:** Charitable Medical Healthcare Foundation, Augusta, GA, United States

**Keywords:** neuroscience mysteries, default space, consciousness, sleep, emotion regulation, binding problem, thalamus, metastability

## Abstract

Aside from the nature of consciousness itself, there are still many unsolved problems in the neurosciences. Despite the vast and quickly growing body of work in this field, we still find ourselves perplexed at seemingly simple qualities of our mental being such as why we need to sleep. The neurosciences are at least beginning to take a hold on these mysteries and are working toward solving them. We hold a perspective that metastable consciousness models, specifically the Default Space Model (DSM), provide insights into these mysteries. In this perspective article, we explore some of these curious questions in order to elucidate the interesting points they bring up. The DSM is a dynamic, global theory of consciousness that involves the maintenance of an internal, 3D simulation of the external, physical world which is the foundation and structure of consciousness. This space is created and filled by multiple frequencies of membrane potential oscillations throughout the brain and body which are organized, synchronized and harmonized by the thalamus. The veracity of the DSM is highlighted here in its ability to further understanding of some of the most puzzling problems in neuroscience.

## Introduction

The most complex object in the known universe is essentially ourselves, our own brains. However, this three-pound mass of buttery consistency is an enigma in that it is “us,” and yet it may seem to be an utterly alien system. Due to its unimaginable complexity, understanding the brain is a very challenging task and there are still many hurdles to overcome before we truly understand our own nature. In order to gain this understanding, we need to ask the right questions. As neuroscientific academia grows, it seems the answers provided for seemingly simple questions become less satisfactory. In this perspective article, we present the perspective that a metastable theory of consciousness which includes physiology of the brain and body is necessary to clarify these questions.

In the neuroscience of consciousness, theories of metastability propose that consciousness arises from the global integration of several functional modules via neural oscillations acting in a cooperative and coordinated manner. In this perspective article, functional modules couple together while still expressing their own innate, independent behavior (Kelso and Tognoli, 2007). Brain-centric metastable models of consciousness such as the Dynamic Core Hypothesis (DCH), Global Workspace Theory (GWT), and the Operational Architectonics Theory of brain-mind (OAT) offer great insight into mysteries of neuroscience, However, these theoretically fall short of the explanatory power provided by a metastable model that includes the brain and body in its theory which is provided by the Default Space Model (DSM; Jerath and Crawford, 2014).

**Figure 1:**
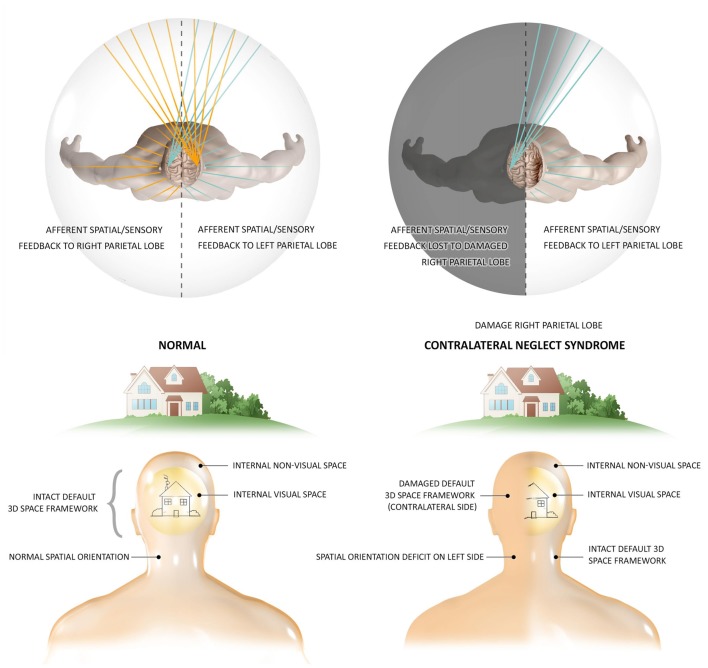
The 3D matrix of consciousness and contralateral neglect syndrome. Contralateral neglect syndrome, resulting from damage to the right parietal lobe, illustrates the independent presence of a subconscious 3D space which is the structural foundation of experience. In the healthy individual who has an intact spatial framework of consciousness, the right parietal lobe maps spatial information on sensory data from both sides of the body, while the left parietal lobe is able to map information from the right side of the body. In this healthy person, sensation from every spatial perspective can be perceived, such as the full image of a house. In patients with contralateral neglect (also termed hemispatial neglect) suffering right parietal lobe damage, information from both sides of the body is fed to this lobe but cannot be spatially mapped to the lobe damage. The intact left parietal lobe can still map information from the right side of the body, but sensation from the left side has no cortex to spatially orient it. These patients not only lack consciousness of such sensory stimuli, but the entire left side of space ceases to consciously exist, illustrating the fundamental necessity of the spatial 3D matrix in the occurrence of consciousness. These patients are not aware of any “missing space,” and experience the remaining right side as the full physical world. The actions of these patients are also restricted to the remaining side, eating food from only one side of their plate and only dressing one side of their body. Research shows that stimuli from the “missing” side is fully processed, but however remains unconscious (Figure by Lynsey Ekema, MSMI. © 2014 Dr. Ravinder Jerath. All rights reserved).

The DCH and GWT both share the DSM’s concept of the thalamocortical system as the functional hub of global module integration (Tononi and Edelman, 1998; Baars et al., 2013; Jerath et al., 2015a), however, the DSM provides a dynamic architectural structure allowing orderly, functional integration which is the conscious, internal, 3D simulation of the external physical environment into a 3D matrix termed the default space. OAT shares this insight into the most fundamental quality of this “virtual reality” of subjective experience, its 3D coordinate system occurring spatio-temporally in tandem with the physical world, correlating this inner reality with temporal and spatial organizations of brain activity (Fingelkurts et al., 2010). This structural foundation of a 3D matrix as the basis of conscious phenomena is illustrated by the neurological condition of contralateral neglect syndrome (Figure 1; Fingelkurts et al., 2010; Jerath and Crawford, 2014). OAT also shares with DSM the engagement of a metaphor of a global, harmonious, musical symphony to describe the electrical oscillatory synchronization of distinct, autonomous neuronal assemblies in the creation of consciousness via large-scale, cooperative, and integrative functions (Fingelkurts et al., 2010; Jerath et al., 2017a). The DSM model however posits that every cell of the body and not just neuronal cells are involved in this oscillatory ensemble which illustrates the holistic nature of consciousness (Jerath et al., 2017a). Although OAT asserts there is a center to this space, DSM takes this view further by describing how the perspective of where the consciousness self is centered in the 3D inner space is based on the spatial location of the thalamus in the brain as all perceptual information is coordinated around it (Jerath et al., 2015a).

DSM and OAT theories propound that functionally integrated, microscopic, mesoscopic and macroscopic bioelectric fields constitute the spatially organized, 3D matrix of consciousness (Fingelkurts et al., 2010; Jerath et al., 2015a). OAT however fails to sufficiently identify biological mechanisms for the distal, macroscopic coordination of global reentrant and oscillatory activity of which DCH, GWT and DSM show with substantial physiological support that the thalamocortical system is responsible for Edelman et al. (2011); Baars et al. (2013); Jerath et al. (2015a). Both DSM and OAT correlate the highly stable oscillatory activity of the default mode network with the conscious sense of self centered in the externalized, perceptual reality of a 3D space (Fingelkurts et al., 2010). The DSM advances these concepts by binding bioelectric operations of the brain such as the default mode network with bioelectric operations of the body such as respiration and cardio activities (Jerath and Crawford, 2015b). The DSM consists of the strongest points of metastable theories of consciousness along with the bioelectric connection of the body and brain which provide the greatest insight into the biggest mysteries of neuroscience.

## Insight Into the Top Mysteries of the Mind

Consciousness is commonly thought to arise from computational processes solely within the brain (Edelman, 2008). Previously, we have reviewed evidential support from studies on respiration physiology and electrophysiology to show that consciousness is a body-wide process and not limited to individual cortical sites (Jerath et al., 2017a) which suggest that it is actually an interactive process between the brain and body. The brain-body theory of the DSM provides insight into current mysteries of the mind and has been supported by physiological research. Through providing insight into unresolved quandaries of neuroscience, we show the veracity of this model and other metastable models and the power they have to improve and understand ourselves.

### What Is the Relationship Between Subjective Experience and the Physical World?

Through optical illusions and other illusions such as the rubber-hand illusion, it is evident that we do not see reality as it truly is, however, we perceive a subjective version of it. This nature is illuminated by the founding notion of the DSM and other “world simulation” models, that the external physical world is subjectively simulated by an internally generated experiential reality (Hesslow, 2002; Revonsuo, 2006; Trehub, 2007). According to the DSM, this internal reality biologically consists of macroscopic, synchronized bioelectric oscillations throughout the brain and body (Jerath et al., 2015a). This bioelectric architecture provides the conscious phenomenon of a 3D matrix framework for the qualities of the external world to be defaulted into (Jerath et al., 2015a). A common human experience is that we directly experience the external world through our senses, however, the DSM explains along with evidence from illusions, neurology and psychology that what we experience is actually heavily influenced by our executive expectations, past experiences, and biases instead of a direct experience of external stimuli (Driver and Vuilleumier, 2001; Smythies, 2003; Grush, 2004; Revonsuo, 2006; Maldonato and Dell’Orco, 2012).

This internal simulation of the external world is therefore a best guess of reality which highlights the aspects that are most important to our tasks at hand which improves survival chances by reducing processing requirements (Maldonato and Dell’Orco, 2012). Both the DSM and OAT suppose that global, multisensory integration and unification into a singular conscious experience is functionally, spatially and temporally reflected in the dynamic structure of the bioelectric fields of the brain (Fingelkurts et al., 2010; and body in the DSM; Jerath et al., 2016). Further research into this mystery should investigate identifying an isomorphism in the space-time of subjective experience with the physical, bioelectric space-time of the brain.

### How Do We So Quickly Process and Interpret the External World?

Of the massive amount of sensory information bombarding our senses at any time, we are only aware of a small percentage, with estimates at 99% of externally derived sensory data being discarded upon entering the brain (Gazzaniga, 1998). The massive amount of computation required to process all of the information inundating our senses at any given time is unnecessary and beyond what the brain is capable of (Kveraga et al., 2007). Computational and theoretical work has shown the top-down predictions and expectations of the mind are integrated with external sensory input to minimize processing demands of perception (Engel et al., 2001). The DSM explains how attention and expectations derived from memory and other cognitive functions synchronize directly with the sensory organs in order to quickly influence what is integrated into the internal replication of the external world (Jerath et al., 2015a).

The bioelectric framework of the default space replicates the external space continuously in time. This space and the “virtual” objects contained within it therefore prime the filling of external sensory stimuli into this space based on expectations derived from the state of the simulation in the previous moment in time (Jerath et al., 2015a). These internal predictions directly influence the sensory organs via oscillatory synchronization with the cortex (Jerath et al., 2016). Through this synchronization, lateral inhibition at a distance influences incoming sensory information so that expected stimuli essentially “fall” into place upon the sensory receptors at which they are predicted through filtering and amplifying, thereby drastically reducing processing required and allowing for increased sensory acuity (Jerath et al., 2017b). Commonly, lateral inhibition indicates cells inhibiting physically adjacent cells. However, in our denotation, cortical inhibitions of sensory organ cells are “lateral” in that they are “adjacent” through synchronization via membrane potential oscillations (Jerath et al., 2016). By bringing the processing power of the cortex to the sensory organs through the synchronization of the current “world simulation” with the sensory organs, the next “frame” of the simulation can be almost immediately updated as the sensory organs are part of the body-wide, bioelectric framework of consciousness (Jerath et al., 2016). Further research may utilize EEG monitoring on the sensory organs and cortex to identify top-down regulation of the sensory organs.

### How Do All of Our Sensations Unify Into One Experience Seamlessly?

The “binding” problem is a mystery of the neurosciences of how a unified conscious experience arises from the distributed activities of the central nervous system (Revonsuo and Newman, 1999). For instance with vision, how is information from thousands of cones in the retina receiving different parts of a visual stimulus integrated into a neural representation of a single object with different characteristics such as form, color, motion, brightness, and depth? Metastable theories such as OAT and the DSM explain how oscillations between distributed modules synchronize together to create the infrastructure of an internal 3D space that functionally binds objects to it and binds sensory information to those objects (Fingelkurts et al., 2010; Jerath et al., 2016). The DSM furthers this notion by asserting the synchrony not only between cortical modules, but also between those cortical modules and the sensory organs (Jerath et al., 2016).

Research has been conducted on exploring the “binding” problem through dynamic oscillations and synchrony which support metastable models (Kelso, 2002; Fingelkurts et al., 2003). There are several neural theories that support the functional importance of top-down mechanisms in synchronistic binding (Engel et al., 2001). The temporal binding model assumes that neural synchrony with precision in the millisecond range is crucial for object representation, and that this synchrony enhances the neural activity important to the task at hand while discarding less valuable activity (Roelfsema et al., 1996). This model predicts that neurons that respond to the same sensory object likely fire in synchrony within the millisecond range and that this synchronization should not exist between neurons that are stimulated by different objects in sensory space (Singer and Gray, 1995). While this supports metastable concepts, the notion of binding is furthered by the DSM not only through the assertion that the sensory receptors are bound to the virtual representations in the 3D default space, but that a harmonious synchrony of oscillations throughout the body and brain provide a united bioelectric structure that creates the internal 3D matrix to which individual modules may bind their perceptual processes to.

Further research may address how global coherence is created while allowing individual cortical models to have their own processing activities and synchronize with other associated modules. This indicates that if total unity/synchronization or total segregation exists among all neural assemblies of the global bioelectric field then a loss of consciousness should occur (Fingelkurts et al., 2010). The main insight metastable models provide to this mystery is that bioelectric synchrony among the processing of distributed assemblies (and sensory organs in the DSM) represent the binding mechanism, and such global binding dynamics leads to the integrated, unified, conscious experience. Patients with conditions that disrupt the abundant, diverse, and complex synchrony among many cortical modules such as schizophrenia (Bressler, 2003; Borisov et al., 2005) should show a disruption in perceptual binding, and this is indeed the case (Stephens and Graham, 2000; Cuesta and Peralta, 2001). Further research should examine other patients with disrupted synchronization to investigate the broadness of this mechanism in perceptual binding.

### Why Do We Sleep?

The function of sleep is still one of the most perplexing mysteries in biology (Frank, 2006). Multiple functions have been proposed such as restoration via the removal of metabolic waste products (Xie et al., 2013), memory consolidation (Walker and Stickgold, 2006), and even energy conservation during dark nights when food cannot be found and safety is an issue (Zepelin et al., 2005). According to the DSM, cardiorespiratory synchronization (Bianchi et al., 2006) and corresponding changes in the autonomic nervous system during slow-wave sleep toward the parasympathetic state (Frostig et al., 1990; Cabiddu et al., 2012) promote the restorative hyperpolarization of neurons throughout the brain (Jerath et al., 2014).

Parasympathetic activation can synchronize multiple physiologic processes, regulating the homeostasis of sleep states (Lurie, 2011; Nasi et al., 2011). Research support for the DSM’s hypothesis on sleep is needed, however, plausibility that increased parasympathetic activity during slow-wave sleep via cardiorespiratory synchronization leads to hyperpolarization of cellular membrane potentials throughout the body is drawn from studies on various cellular membrane potentials during prolonged parasympathetic activation (Sato et al., 2006; Hanson et al., 2012). Additionally, studies have demonstrated hyperpolarization of neural membrane potentials during slow-wave sleep (Hirsch et al., 1983; Steriade et al., 2001) and that sleep-deprived brains show increased membrane excitability (Winters et al., 2011; Yan et al., 2011). The restorative function of membrane hyperpolarization via oscillations may work by combating overly excitable membranes that may disturb action potential precision and stimulus discrimination (Schaefer et al., 2006). Further research should investigate the nature of global membrane hyperpolarization during sleep and how this may provide a restorative function.

### How and By What Mechanisms Can Emotions Be Regulated?

Negative emotions and psychological stress have been shown to play significant roles in the development of health problems such as hypertension and coronary heart disease (Treiber et al., 2001; Snieder et al., 2002; Todaro et al., 2003). By what methods can we learn to accept our negative emotions and move toward a more positive mental atmosphere? Under stress, a sympathetic response occurs and cardiorespiratory synchronization decreases (Zhang et al., 2010). The parasympathetic state has been identified as a predictor for the success of instructed mood repair (Yaroslavsky et al., 2016). In modern society, our autonomic nervous systems, evolutionary developed for a much different environment, are often chronically stuck in the stress-induced “fight or flight” response of the sympathetic nervous system (Nesse et al., 2016). Although our ancestors lives were likely more stressful overall, the sympathetic nervous system was activated only when physical danger was present, whereas today, our tendencies to commit ourselves to goals and responsibilities we cannot achieve in part leads the sympathetic response to be activated for greatly extended periods (Nesse et al., 2016).

**Figure 2:**
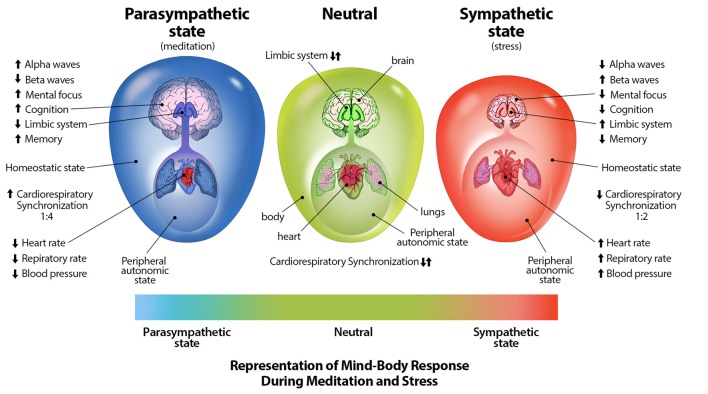
Representation of mind-body techniques on the autonomic nervous system. This figure correlates a spectrum of emotional states with a spectrum of physiological states. The state of distress is correlated with the sympathetic state of the autonomic nervous system. The large outer oval represents the homeostatic state of the body, while the size the large inner oval represents a chaotic-calm spectrum of bodily afferent signaling. The body-brain connection is stronger in the parasympathetic state illustrating increased synchronization and coherence. The size of the brain represents overall cognitive ability and the level of control it exerts on overall homeostasis. Other indications in this image further illustrate the benefits of parasympathic dominace which can be cultured through meditative techniques (Figure by Michael Jensen MSMI, CMI).

Through mind-body interventions such as mindfulness and meditative breathing exercises in which a synchrony between cardiac and respiratory rates is created, a parasympathetic response (Cysarz and Büssing, 2005; Tang et al., 2009; Chang and Lo, 2013), as well as inhibition of amygdala activity (Creswell et al., 2007) which is stimulated during distress (Davis, 1992), can occur. Through practice of such breathing techniques, one may be able to reduce anxiety, distress, neuroticism, and emotional disorders, and increase positive well-being (Jerath et al., 2006, 2015b; Rosenkranz et al., 2013; Goyal et al., 2014). The physiologically supported perspective of the DSM on the autonomic nervous system and its relation to distress can be illustrated graphically (Figure 2; Jerath et al., 2015a).

Through intentional modification of one’s afferent signaling, the nervous system can be regulated in a way that leads to regulation of emotion (Jerath and Crawford, 2015a). This assertion is made from research indicating that internal feedback from physiological changes influence emotional states (Critchley, 2005). Although breathing may be the most powerful, or at least most studied mechanisms of affective change, other forms of affective signaling modification have shown to be effective in emotional regulation. For instance, forced facial expressions can result in the emotion indicated by the facial expression. Forced smiling and laughter has been shown to significantly improve mood in participants (Neuhoff and Schaefer, 2002). Other non-respiratory mediation techniques have been shown to induce positive affective change. For instance, Transcendental Meditation, which does not involve breath control, has been indicated to reduce sympathetic tone and stress reactivity (Barnes and Orme-Johnson, 2012).

Depending on the breathing technique, the autonomic nervous system can be voluntarily influenced in different ways. Cyclic hyperventilation followed by breath retention has been demonstrated to result in activation of the sympathetic nervous system (Kox et al., 2014). Sympathetic, “flight or fight,” arousal states are associated with rapid breathing (Nyklíček et al., 1997). Such respiratory rhythms are generated in regions of the brainstem (Feldman and Del Negro, 2006) which illustrate the connection between respiration and emotion as these regions have reentrant connections with the limbic system (Evans, 2010). Cardiovascular afferent signaling has also been shown to evoke autonomic responses (Gray et al., 2012). This research has formed a basis for the proposition that the phase synchronization of the heartbeat with respiration (cardiorespiratory coherence) can have a greatly expanded effect on the autonomic nervous system (Jerath and Crawford, 2015a). Through the practice of meditative and deep breathing techniques, one can modulate visceral afferent signaling which in turn can modulate the autonomic nervous system, brainstem, and cortical areas of the brain leading to improvements in anxiety, chronic stress, and mood disorders (Jerath and Crawford, 2015a). Although a great deal of research has been conducted on stress and meditation, further research into meditation may yield greater insights by focusing on mind-body synchronization and the effects of generating parasympathetic dominance.

## Conclusion

The neurosciences have made tremendous progress in deciphering the activities of almost 100 billion neurons in a single human brain, however there are many more mysteries that await elucidation. In light of metastable models of consciousness such as the DSM, we have provided insight into some of the more tantalizing questions that mystify neuroscientists. Although some of these questions may seem obvious, current scientific understanding of their essence is limited. The insights we have provided illustrate the veracity of the DSM and the power of metastable models in illuminating the shadows of neuroscience.

## Author Contributions

RJ has developed the theory with some contribution to the writing of the manuscript. CB has made the major contribution in the writing of the manuscript.

## Conflict of Interest Statement

The authors declare that the research was conducted in the absence of any commercial or financial relationships that could be construed as a potential conflict of interest.
